# Supplementary material for: Dose-Dependent Sorafenib-Induced Immunosuppression Is Associated with Aberrant NFAT Activation and Expression of PD-1 in T Cells
Source: Cancers (Basel). 2019 May 16;11(5):681. doi: 10.3390/cancers11050681 (PMC6562672; doi:10.3390/cancers11050681)
Supplement: Supplementary file 1 [file cancers-11-00681-s001.pdf]

Supplementary Materials: Dose-Dependent Sorafenib-Induced Immunosuppression Is Associated with Aberrant NFAT Activation and Expression of PD-1 in T Cells

Renuka V. Iyer, Orla Maguire, Minhyung Kim, Leslie I. Curtin, Sandra Sexton, Daniel T. Fisher, Sarah A. Schihl, Gerald Fetterly, Stephan Menne and Hans Minderman

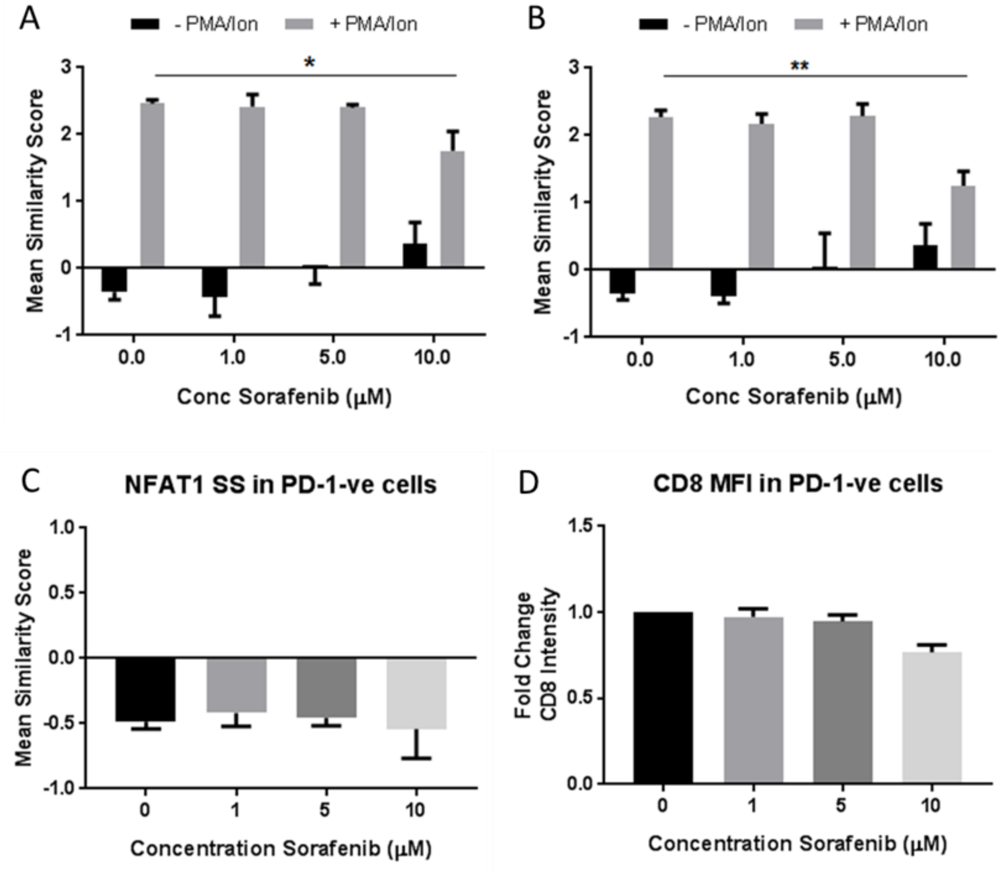

Figure S1. Sorafenib has dose-dependent effects on NFAT nuclear localization in T cells. (**A**) NFAT1 nuclear localization, measured by mean similarity score by imaging flow cytometry, exhibits a dose-dependent increase without TCR stimulation (-PMA/Ionomycin), and decreases with PMA/Ionomycin stimulation in CD8+ T cells, and (**B**) in CD4+T cells. (**C**) NFAT1 similarity score does not change in CD8+ T cells that are PD-1 negative, (**D**) Graph shows that CD8 expression measured as a fold change from the untreated sample (black bar) does not significantly change in PD-1- CD8+ T cells. Experiments are $n = 3$, *; $p < 0.05$; **; $p < 0.01$, by Student's t-test.

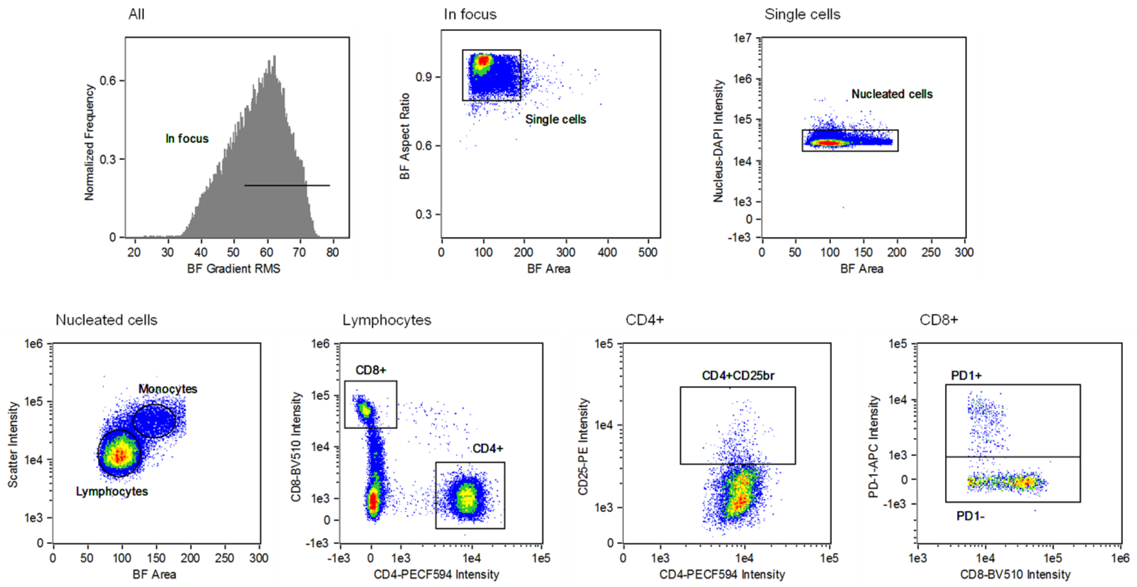

Figure S2. Hierarchical gating strategy used to analyze T cell sub-set expression and NFAT1 nuclear translocation Upper graphs from left to right: Events which are in focus are selected on the basis of a high value of a contrast parameter of the brightfield image (BF gradient RMS). Of those cells, single cells are discriminated from debris and cell aggregates based on area and aspect ratio of the brightfield image. Single, in focus cells that are nucleated are then selected. Lower graphs from left to right: Mononuclear cells are separated into lymphocytes and monocytes by area and scatter properties. Lymphocytes were then further phenotypically separated into CD4+ and CD8+ T cells. CD4+ T cells that were positive for CD25 were selected. For PD-1 expression experiments the PD-1 expression in both CD8+ cells (shown) and in CD4+ cells was graphed.

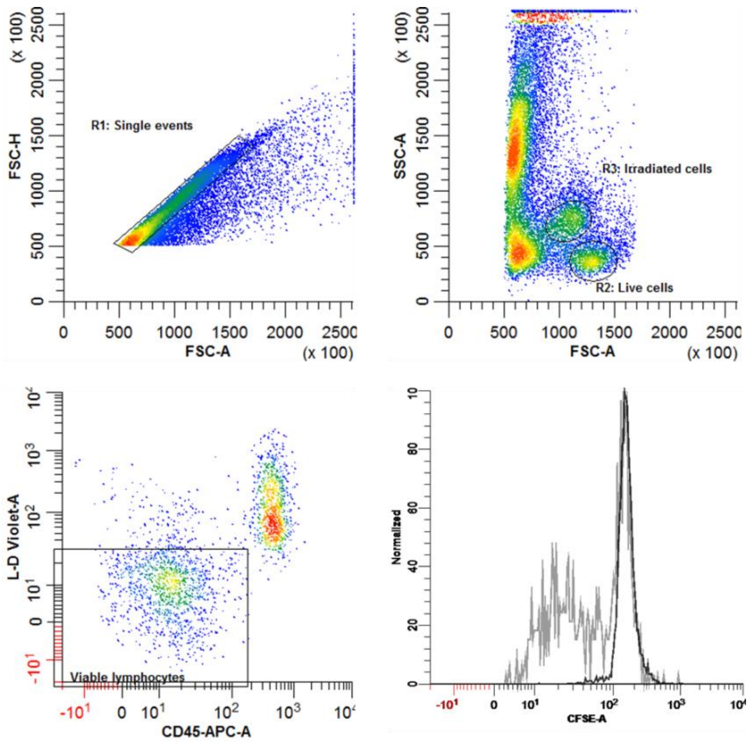

Figure S3. Hierarchical gating strategy used in Mixed Lymphocyte Reaction (MLR) analysis. Single events are selected based of forward scatter area versus height. Responder cells (Live cells) are then separated from irradiated cells by forward scatter versus side scatter. Viable responder cells are further selected on the basis of being negative for LiveDead Violet and negative for CD45-APC used to stain irradiated stimulator cells. Percent proliferation of responder cells measured in relation to non-proliferating cells (black histogram). Cells with a lower CFSE intensity are proliferating.

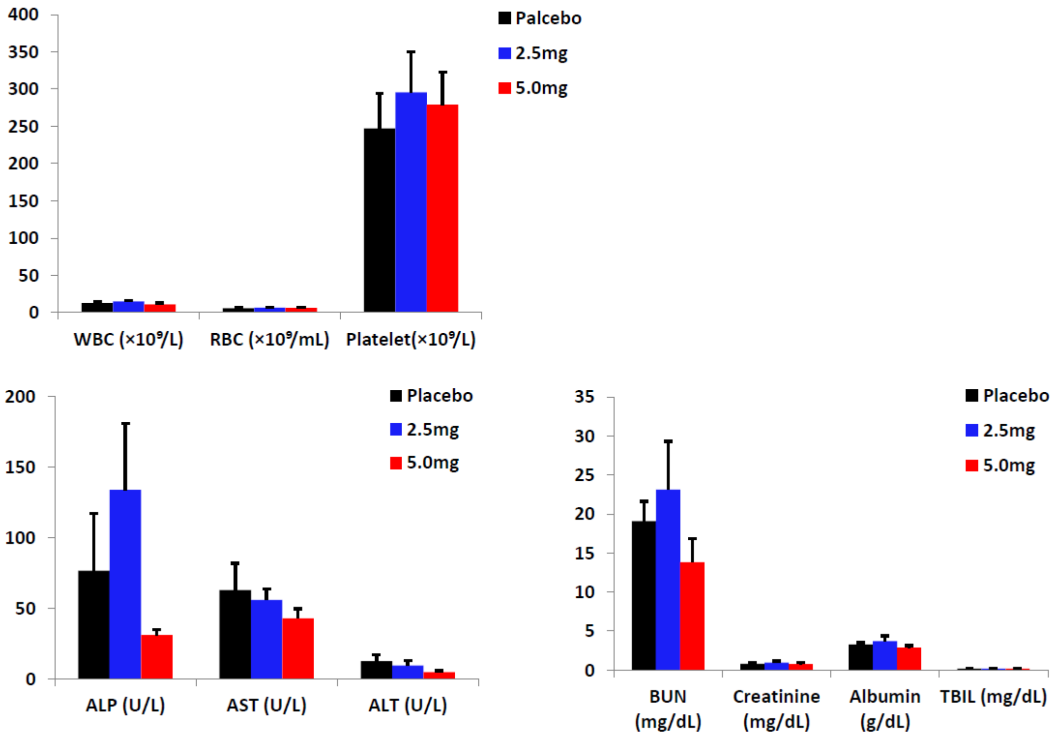

Figure S4. CBC and biochemistry analyses in blood at the end point of experiments.

Table S1. List of antibodies and dyes used in flow cytometry and imaging flow cytometry experiments.

Antigen	Fluor	Clone	Vendor
CD45	APC	2D1	BD Biosciences
CD4	PECF594	RPA-T4	BD Biosciences
CD25	PE	2A3	BD Biosciences
CD8	BV510	RPA-T8	BioLegend
CD3	PE	G4.18	BD Biosciences
CD279 (PD-1)	APC	MIH4	BD Biosciences
NFAT1	Unconjugated	D43B1	Cell Signaling
Dky Anti Rb 2Ab	AF647	NA	Jackson ImmunoResearch
Dky Anti Rb 2Ab	FITC	NA	Jackson ImmunoResearch
DAPI	NA	NA	ThermoFisher
CFSE	NA	NA	ThermoFisher

© 2019 by the authors. Licensee MDPI, Basel, Switzerland. This article is an open access article distributed under the terms and conditions of the Creative Commons Attribution (CC BY) license (http://creativecommons.org/licenses/by/4.0/).
